# Multivariate modelling with ^1^H NMR of pleural effusion in murine cerebral malaria

**DOI:** 10.1186/1475-2875-10-330

**Published:** 2011-11-02

**Authors:** Soumita Ghosh, Arjun Sengupta, Shobhona Sharma, Haripalsingh M Sonawat

**Affiliations:** 1 Department of Chemical Sciences, Tata Institute of Fundamental Research, 1-Homi Bhabha Road, Mumbai, Mumbai-400005.India; 2 Department of Biological Sciences, Tata Institute of Fundamental Research, 1-Homi Bhabha Road, Mumbai-400 005, India

**Keywords:** Cerebral Malaria, *Plasmodium berghei *ANKA, pleural effusion, NMR, Orthogonal Partial Least Square Discriminant Analysis, Multiway Principal Component Analysis, Multivariate Curve Resolution

## Abstract

**Background:**

Cerebral malaria is a clinical manifestation of *Plasmodium falciparum *infection. Although brain damage is the predominant pathophysiological complication of cerebral malaria (CM), respiratory distress, acute lung injury, hydrothorax/pleural effusion are also observed in several cases. Immunological parameters have been assessed in pleural fluid in murine models; however there are no reports of characterization of metabolites present in pleural effusion.

**Methods:**

^1^H NMR of the sera and the pleural effusion of cerebral malaria infected mice were analyzed using principal component analysis, orthogonal partial least square analysis, multiway principal component analysis, and multivariate curve resolution.

**Results:**

It has been observed that there was 100% occurrence of pleural effusion (PE) in the mice affected with CM, as opposed to those are non-cerebral and succumbing to hyperparasitaemia (NCM/HP). An analysis of ^1^H NMR and SDS-PAGE profile of PE and serum samples of each of the CM mice exhibited a similar profile in terms of constituents. Multivariate analysis on these two classes of biofluids was performed and significant differences were detected in concentrations of metabolites. Glucose, creatine and glutamine contents were high in the PE and lipids being high in the sera. Multivariate curve resolution between sera and pleural effusion showed that changes in PE co-varied with that of serum in CM mice. The increase of glucose in PE is negatively correlated to the glucose in serum in CM as obtained from the result of multiway principal component analysis.

**Conclusions:**

This study reports for the first time, the characterization of metabolites in pleural effusion formed during murine cerebral malaria. The study indicates that the origin of PE metabolites in murine CM may be the serum. The loss of the components like glucose, glutamine and creatine into the PE may worsen the situation of patients, in conjunction with the enhanced glycolysis, glutaminolysis and increased activity of creatine phophokinase which are already reported characteristic pathophysiological features of malaria.

## Background

Malaria is a serious disease which is widespread in tropical and subtropical countries, causing the death of about one million per year [1]. Nearly 40% of the world population is under risk of malaria. One of the major complications of the disease is cerebral malaria, caused in humans by the unicellular protozoan *Plasmodium falciparum*. The intraerytrocytic stages of the parasite are responsible for much of the clinical manifestation of the disease [2,3]. Cerebral malaria is accompanied by various disorders and complications such as high fever, anaemia, haemoglobinuria [4], retinal damage [5] ALI/ARDS [6], pulmonary oedema [7-10] and pleural effusion [11-13]. ARDS was reported as an important predictor of mortality in adults due to malaria [9] and is associated with a greater than 70% fatality rate. In experimental falciparum and vivax malaria, it was observed that Aotus monkeys showed no symptoms till 72 hours before death ascribed to pulmonary oedema and pleural effusion [14]. While pulmonary involvement is a recognized complication of malaria infection, little is currently known about its pathogenesis [15]. Pulmonary oedema and pleural effusion are thus important aspects to be investigated during this disease. Some of the clinical and pathologic aspects are known for these complications; like increased vascular permeability [16], vasodilatation that might lead to cardiac insufficiency [17]. The increase in the vascular permeability and decrease in osmotic pressure of serum colloid play a crucial role in circulatory shock in cerebral malaria [18]. Circulatory shock is a pathophysiologic state in which tissue perfusion is totally inadequate to meet oxygen or nutritional needs of the cells. Circulatory shock is also known as septic shock and is characterized by vasodilatation, decrease in arterial pressure and changes in the vascular permeability [19].

The murine model of the disease provides a convenient alternative for investigating changes in biofluids during the disease progression to cerebral malaria. The model consists of *Plasmodium berghei *(ANKA strain) as the causative agent used in combination with C57BL/6 mice [20] which shares many symptoms and features of human CM [21]. This mouse model has been used in studies of malarial lung syndromes [22,23]. The vascular permeability is known to be increased in brain, lungs, kidneys and heart in murine model of CM [24]. The vascular permeability and immune parameters have been studied for murine malaria [25], but no report exists on the metabolite composition of the PE.

^1^H NMR spectroscopy coupled with multivariate statistics is used to understand perturbation of metabolism in various diseases. It is an unbiased technique as it gives spectral profile of all the metabolites present. This method has been used to understand disease condition such as breast cancer [26], malaria [27] diabetes [28] and coronary heart disease [29] and many other diseases.

In this study, the PE that is produced in cerebral malaria has been characterized and the profile was compared with serum for each mouse by ^1^H NMR spectroscopy, followed by multivariate statistical analysis. The study indicates that although PE comes from serum there are differences in concentrations of some metabolites in these two biofluids. A multiway PCA (MPCA) analysis and MCR-ALS study of the two biofluids were used to assess the metabolic correlations across these fluids and the contribution of one compartment over the other respectively. MCR-ALS also provided a pure spectrotype of each compartment. The pure spectrotype of the pleural effusion is dominated by glucose and that both the compartments have contributions towards each other.

## Methods

### Animal handling

All the animals were treated in accordance with the guidelines set forth by the local animal ethics committee of TIFR.

### Animal experiments

25 female C57BL/6 mice of age 6 to 8 weeks and weight (24-25 gm) were housed in five cages (5 mice in each cage). They had free access to standard food pallets and water. The temperature was maintained at 22 ± 2°C and they were kept under 12 hour day night cycle. Twenty of these mice were injected inrtaperitonially with 10^7 ^RBCs each infected with *P. berghei *ANKA. The remaining five mice served as uninfected controls. These parasites were maintained in Swiss female mice of 6 to 8 weeks. Rectal temperature of all the mice was measured twice a day with a digital thermometer.

Parasitaemia was monitored daily by counting number of infected cells per 1,000 RBCs in Giemsa-stained [30] slides of blood smear from tail bleeds. The mice were considered to have CM if they displayed one or more of the neurological symptoms such as ataxia, paralysis, convulsions, deviation of head etc or had body temperature < 34°C [31,32]. When the prominent symptoms of the CM were visible on day 9 post-infection CM mice were anesthetized with ether and blood was collected by retro-orbital bleeding. The mice were dissected after this and the pleural fluid was collected by aspiration. The collection of the two biofluids (PE and sera) from two different regions of the body of CM mice ensures no mixing of the fluids. The PE collected from the mice was colorless. Among 10 mice that showed symptoms of CM, one mouse died during the course of the experiment and one exhibited too small a volume of PE compared to other CM mice, so their PE is not used for the experiment. The remaining eight animals were used for the study. The amount of fluid varied from 200 to 400 μl. The mice that did not exhibit neurological symptoms or a high body temperature > 34°C were considered as the non-CM (NCM) mice. The parasitaemia of the CM mice rose to 18 ± 2% during the time of sacrifice. The parasitaemia of NCM mice were comparable. In the control and NCM mice pleural fluid was collected by protocol mentioned in [33]. Briefly, the mouse was anaesthetized, a 30 μl gel loading tip was inserted through an inter-coastal space and the pleural fluid was withdrawn. In some animals 100 μl of 0.075 M phosphate buffer pH 7.4 was injected into the pleural cavity of the animal and after waiting for a minute the fluid was withdrawn.

### Sample preparations for NMR spectroscopy

The blood samples as well as the PE and pleural fluid were incubated in 37°C for 10 mins and were centrifuged for 10 mins at 13100 g. The supernatant was collected, frozen in liquid N_2 _and stored at -80°C. For NMR experiments, 180 μl of sample (sera or pleural effusion) was mixed with 400 μl of the pH 7.4 buffer containing 0.075 M Na_2_HPO_4._7H_2_0, 4% NaN_3 _and 0.02% DSS, 20%D_2_O (the buffer recipes were provided by Bruker Biospin Metabonomic unit). In case of pleural fluid of NCM the final volume of the sample was maintained to 580 μl by adding 0.075 M phosphate buffer pH 7.4. The samples were placed in 5 mm NMR tube (Norrell Inc.).

### ^1^H NMR spectroscopy

One dimensional ^1^H NMR spectra were acquired on AVANCE 700 MHz Bruker spectrometer with triple resonance probe using D_2_O as the frequency lock at 310 K. The pulse sequence used has the form -RD-90°-(t-180°-t)_n_-ACQ with presaturation at the water peak. Here RD implies a relaxation delay of 4 s; no of loop (n) being 128 and CPMG spin echo time (t used was 300 μs). The purpose of using CPMG spin echo is to attenuate the broad signals from the macromolecules that may be present in the sample. A total of 32 transients were collected into 88640 data points using a spectral width of 20.059 ppm. For pleural fluid of NCM the number of transients was increased to 512. The FID was subjected to exponential multiplication leading to an additional line broadening of 1 Hz, a Gaussian multiplication of 0.01 Hz and a sine squared bell apodization function prior to Fourier transformation. Spectra were phased and baseline corrected using the protocols provided by Bruker Biospin. The assignments of metabolites were based on 2D COSY and TOCSY spectra. In addition, human metabolome database [34] was also used for this purpose. For COSY experiments, 64 transients per increment and 256 increments were collected in direct and indirect dimension respectively. A QSINE function with 2048 and 1024 digital points were used for processing. Exponential multiplication of 0.20 and 0.30 Hz in the direct and indirect dimension respectively was used. TOCSY was processed using SINE function. Exponential multiplication of 0.20 and 0.30 Hz in the direct and indirect dimension was used respectively. In addition, a Gaussian multiplication of 0.1 Hz in the indirect dimension was applied for the TOCSY.

### Data pre-processing

Spectral region of 0.5 to 4.14 ppm was bucketed into frequency window of 0.003 ppm. The region corresponding to water (4.5 to 5.5 ppm) was excluded during binning to avoid artifacts due to pre-saturation of water. The aromatic region was also excluded because the signal to noise ratio in this region was poorer compared to that of aliphatic region. The peaks (1.15(t), 3.35(q)) corresponding to ether were removed before binning. The resulting integrals were normalized to the working region (0.5-4.14) ppm of the spectrum to correct for inter-sample differences in dilution. The binning and the normalizations were achieved using AMIX 2.0 software. The matrix obtained in AMIX was imported to SIMCA P+ 12.0 (Umetrics AB, Sweden) and Solo 6.0 (eigenvector research incorporated software) for further multivariate data analyses.

### Multivariate data analysis

#### Principal component analysis and orthogonal partial least square discriminant analysis

The 2D data matrix was subjected to multivariate statistical analyses. First step of the analysis was a Principal Component Analysis (PCA) [35]. This is an unsupervised method, which gives any overall trend in the data. PCA was conducted for this purpose and to see if there were any outliers. This was followed by Orthogonal Partial Least Square Discriminant Analysis (OPLS-DA) which is a supervised method [36]. OPLS-DA gives segregation between two classes along the predictive component. The R^2 ^(cum) and Q^2 ^(cum) are the two parameters that judge the model. The former explains the total variations in the data while the latter is a cross validation parameter, which indicates the predictability of the model. A 7^th ^of the observations are excluded in each cross-validated round. The processed data is visualized as 2D scores plot. The difference in the relative concentrations of metabolites in serum and PE are interpreted by OPLS coefficient plot [37]. In this, the OPLS modelled covariance, [cov (t_p_, X)], is plotted against the chemical shift and the plot is colored with OPLS modelled correlation [cor (t_p_, X)] also known as p(corr). This plot was generated using a script developed in-house in GNUPLOT 4.4. The orientation of the peaks signifies relative concentration of metabolite in serum and PE of the mice. The signals that point upwards in OPLS coefficient plot indicate a relatively higher concentration of the metabolites in serum with respect to PE during CM whereas a signal pointing downwards indicates a relatively higher concentration of the corresponding metabolites in the PE with respect to serum. The colour of the signals in the plot signifies the contribution of the metabolites towards class segregation, PE and sera. The colour black signifies no significance with the class entities whereas colour yellow signifies highest significance. A color bar associated to the plot indicates the correlation of the metabolites in segregating between classes.

#### Multivariate curve resolution-alternating least squares (MCR-ALS)

MCR-ALS provides a bilinear decomposition of the data matrix into a set of factors [38-40]. These are expressed as pure contributions C (contribution profiles) and S (spectral variables). In equation 1, Xcw corresponds to the ^1^H NMR spectra of the sera and the PE augmented column-wise. Concentration profiles C reflect changes in the contribution of different compartment to each factor. The term S define spectral feature of each factor. The data can be structured in many ways before the algorithm could be applied like column-wise (CW augmentation), row-wise, and row-wise and column-wise augmentation. In this study, CW augmentation was applied. According to this ordering MCR model is described as below

(1) $$\text{Xcw}=\left(\begin{array}{c} x_{1}\\ ∙\\ ∙\\ x_{N} \end{array}\right)=\left(\begin{array}{c} C_{1}\\ ∙\\ ∙\\ C_{N} \end{array}\right){\text{S}}^{\text{T}}+\left(\begin{array}{c} E_{1}\\ ∙\\ ∙\\ E_{N} \end{array}\right)$$

The fitting of the model was done with non-negativity constraints in both the modes. Non-negative constraints were applied using non-negative least squares to both, the contribution and spectral profiles. PCA on the CW augmented matrix was done to choose for the suitable number of factors. The spectral profiles were normalized to unit length before application of MCR-ALS algorithm. For each factor contribution profile shows the distribution of individual metabolic profiles in accordance to the biochemical composition of the compartments. The analysis of the MCR-ALS is done using Solo 6.0 (eigenvector research incorporated software). The assignments of the peaks in MCR are in accordance to ^1^H CPMG spectrum of the corresponding compartment.

### Multiway PCA (MPCA)

MPCA helps us to understand the metabolic correlations between different organs and compartments of the body [41]. Since the two biofluids, serum and PE are present in two different compartments, MPCA would give metabolic correlations that could happen across these two compartments. MPCA will give us information of how the PE is correlated to the changes in the sera and vice versa during CM. MPCA is an extension of PCA and is used for higher dimension of data matrix. Here the N order data are decomposed into scores (t) and loadings (p) and the residual matrix (E). The N order of the data corresponds to number of compartments, which is two in this case; PE and serum. The residuals are considered to the non-deterministic part of the data matrix. MPCA models were prepared, to study the metabolic correlation of PE and serum. A 3D matrix was prepared in Matlab 7.0.1 where the first axis corresponds to spectral variables, the second axis corresponds to compartments (serum, PE) and the third corresponds to individual animals. The loading matrix contains the information of second and third mode [38]. The analysis of the MPCA is done using Solo 6.0 (eigenvector research incorporated software). The peaks in MPCA loading plot were assigned in accordance to the resonances in real ^1^H CPMG spectrum of corresponding compartment.

### Glucose quantification

Glucose quantification in all the sera and PE samples of CM mice and crude pleural fluid of uninfected C57BL/6 were done by glucose oxidase/peroxidase method using glucose quantification kit (Siemens) [42]. Briefly, 10 μL of the sample (serum or PE) was mixed with 1 ml of the "working solution" (95 mmol phosphate buffer, 0.3 mmol 4-aminoantipyrine, 5.9 mmol p-hydroxy benzoic acid, glucose oxidase ≥(5000 U/L), peroxidase ≥ (50000 U/L). The samples were incubated for 15 mins at 37°C. The intensity of the color was measured at 505 nm wavelength in UV-VIS spectrophotometer (Specord, Analytikjena). The concentration of glucose was calculated for all the samples and the significance was checked by Mann Whitney U test.

### Quantification of creatine, glutamine and lipids from the normalized ^1^H NMR spectrum

Relative levels of creatine (3.03 ppm; singlet) and glutamine (2.44 ppm; multiplet) and lipids (1.27 ppm; broad hump) were estimated for both sera and PE from the integrals of the corresponding resonances of the normalized ^1^H NMR spectra. The significance of levels of metabolites in the two biofluids (sera and pleural effusion) was checked by Mann Whitney U Test.

## Results and discussion

### Transition of non-cerebral malaria into cerebral malaria

**Figure 1:**
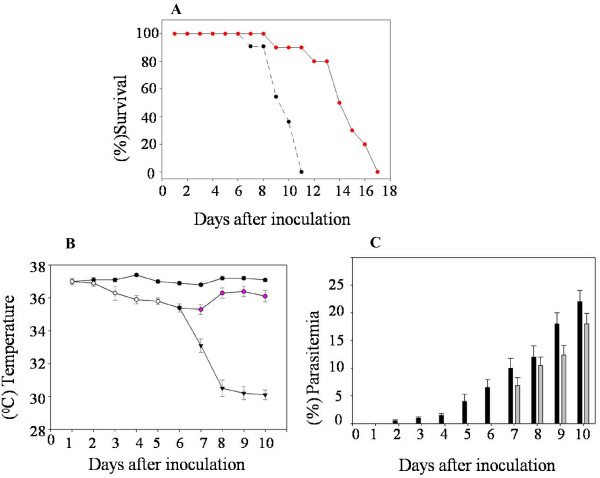
**Parameters of C57BL/6 mice monitored post-inoculation with RBCs infected with *Plasmodium berghei *ANKA**. (A)% Survival of the NCM mice (red circles) and CM mice (black circles) post-inoculation with parasite. (B) The rectal temperature monitored in the uninfected controls (filled black circles) and the PbA inoculated animals (open circles). The NCM mice are pink circles while the CM mice are black triangles. (C) The PbA infected animals are represented by black bars till day 6. After day 6 the black bar refers to the CM and the grey bar graph to the NCM mice.

C57BL/6 mice infected with malarial parasite *P. berghei *ANKA is a well established mouse model of experimental CM [43,44]. The fractions of infected mice that transit into CM exhibit variation [16,43,45]. In our experiments we monitored this combination of mice and parasite over the time of disease progression. During several such trials a consistent observation was that 40-60% of the animals exhibited symptoms of CM. The grouping of a mouse as having CM, versus non-cerebral malaria (NCM) was based on daily measurement of rectal temperature and parasitaemia post-infection (p.i.) (Figure 1). In addition, clinical symptoms including ataxia, paralysis, coma and death were also recorded. Upon dissection on day 9 p.i. we observed that all mice of CM category showed collection of fluid in the pleural cavity. None of either the NCM or uninfected mice showed any pleural effusion (PE). It was therefore, decided to explore this phenomenon further and compared the constituents of serum and the PE of the CM mice.

### ^1^H NMR spectroscopic analysis of serum pleural effusion and pleural fluid

**Figure 2:**
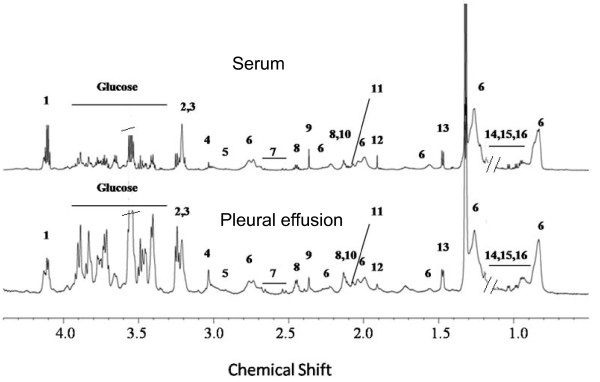
**Representative 700 MHz ^1^H CPMG NMR spectra of serum and pleural effusion of a CM C57BL/6 female mouse**. 1-Lactate, 2-GPC/PC, 3-choline, 4-creatine, 5-lysine, 6-lipids, 7-citrate, 8-glutamine, 9-pyruvate, 10-glutamate, 11-methionine, 12-acetate, 13-alanine, 14-leucine, 15-isoleucine, 16-valine. The broken lines indicate the removed triplet of ether whereas the quartet of ether is marked as red.

**Figure 3:**
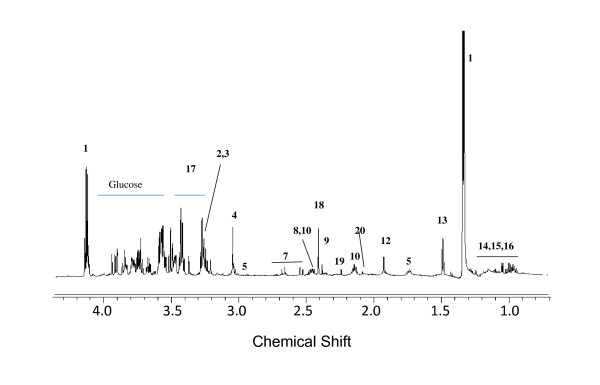
**Representative 700 MHz ^1^H CPMG NMR spectra of pleural fluid of a NCM C57BL/6 female mouse**. 1-Lactate, 2,3-choline/PC, 4-creatine, 5-lysine, 7-citrate, 8-glutamine, 9-pyruvate, 10-glutamate, 12-acetate, 13-alanine, 14-leucine, 15-isoleucine,16-valine, 17-taurine,18-succinate,19-acetone, 20-acetylglycine.

**Figure 4:**
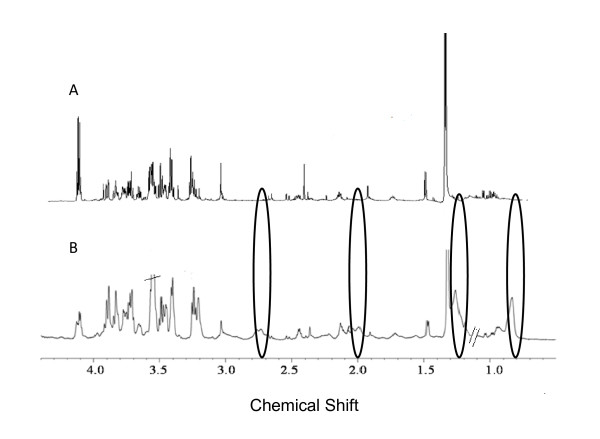
**Comparison of 700 MHz CPMG ^1^H NMR spectrum of (A) Pleural fluid of NCM and (B) Pleural effusion of CM**. The black elipses denote the lipid peaks which are present in CM pleural effusion and absent in NCM pleural fluid.

The ^1^H NMR spectra of the PE and serum of mice that transited into CM are shown in Figure 2 while that of the pleural fluid of the NCM mouse is shown in Figure 3. The assignment of the resonances was achieved by a combination of database search [34] and 2D-Correlated spectroscopy (COSY) and 2D-Total Correlated spectroscopy (TOCSY) experiments, and it was apparent that the constituents of the two biofluids were similar. A comparison of the SDS-PAGE of the serum and PE were similar as well (Additional file 1). The high similarity in SDS-PAGE profile and ^1^H NMR profile of the PE with that of the sera indicates that there is transfer of the serum from the blood vessels resulting in the accumulation of PE. In this context, it is also important to note that there are differences in the ^1^H NMR profile of pleural fluid of NCM and pleural effusion of CM as seen in Figure 4. The prominent differences appear in the absence of lipid peaks in pleural fluid. This is also evident from the multivariate analysis in which we see a high concentration of lipids in CM PE and high concentration of lactate in NCM pleural fluid (Table 1).

**Table 1:** OPLS-DA loadings [(p(1)] and the correlation p(corr) of significant metabolites towards class segregation between PE of CM and pleural fluid of NCM

Metabolites	Resonances	P[(1)]	P(corr)
Lipids (↑CM PE)	1.25, 2.02, 0.83	-0.06, -0.02, -0.06	-0.90, -0.91,-0.93
Lactate (↓CM PE)	1.33	0.27	0.95

### Multivariate modelling of the metabolic profiles of serum and PE in CM

**Figure 5:**
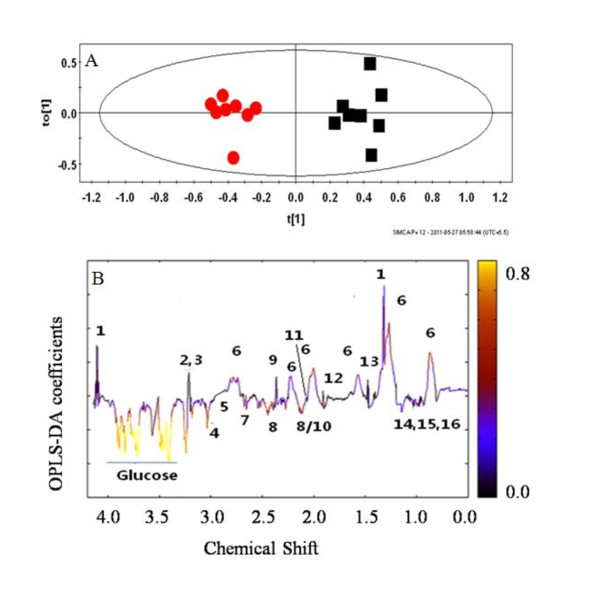
**OPLS-DA scores plot of metabolite profiles based on ^1^H NMR spectra of sera and pleural effusion of C57BL/6 mice with CM, and OPLS-DA coefficient plot of pleural effusion and sera of CM**. The key to the peaks are given in Figure 2. (A) Each red circles denotes score of one ^1^H CPMG NMR of pleural effusion and the black circle denotes score of one ^1^H CPMG NMR of serum of CM C57BL/6 mouse at day 9 p.i in an OPLS-DA analysis. The ellipse is a 95% Hotelling T^2^. R2X (cum) = 0.76, Q2 (cum) = 0.85. (B). The color of the signals in this plot signifies the contribution of the metabolites towards class segregation between PE and sera of CM C57BL/6. A color bar associated to the plot indicates the correlation of the metabolites in segregating between classes (CM sera and CM pleural effusion) with least significance of color black and highest significance of yellow color. The upward orientation of the peaks denotes relatively higher concentration of the corresponding metabolites in sera and vice-versa.

**Figure 6:**
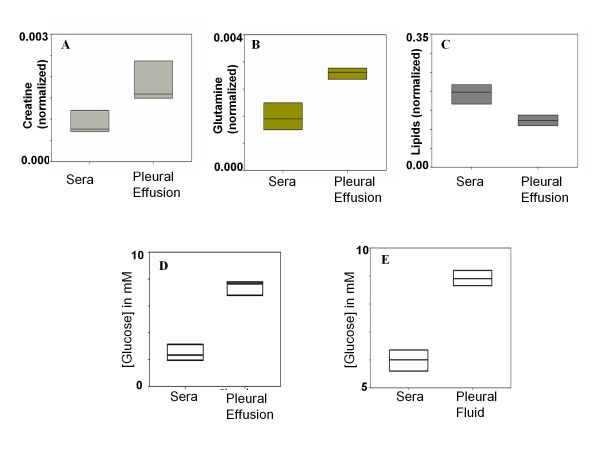
**Relative levels of metabolites in sera and pleural effusion of CM C57BL/6 mice**. (A) Creatine, (B) glutamine, (C) lipids, (D) absolute levels of glucose in mmols/lit in sera and pleural effusion, and (E) absolute concentration of glucose in mmols/lit in sera and pleural fluid of uninfected control mice.

**Figure 7:**
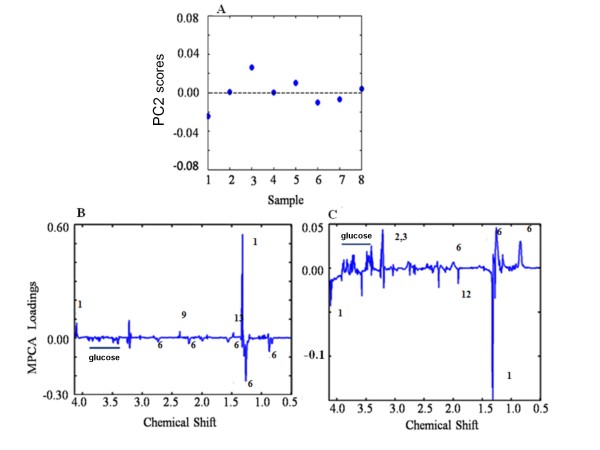
**MPCA scores plot based on CPMG ^1^H NMR spectra of sera and pleural effusion of C57BL/6 mice with CM on PC 2 and MPCA loadings plot of CPMG ^1^H NMR of sera and pleural effusion of CM C57BL/6 mice**. The numbers assigned to the peaks corresponds to the numbers in Figure 2. (A) Each blue dot represents score of a CM mouse (considering sera and pleural effusion together) on PC2 in a MPCA plot. (B) Loadings line plot of sera and (C) pleural effusion of CM mice on PC2 of MPCA. The opposite direction of the signals bears a meaning of anticorrelations among the corresponding metabolites whereas similar direction of the signals indicates positive correlation among the metabolites.

To begin with, the serum and PE of the CM mice were compared using PCA and it was expected that equilibrium would prevail between these two biofluids since the constituents of the two biofluids exhibited by ^1^H NMR spectra and the SDS-PAGE are similar. The PCA scores plot (Additional file 2) represents a NMR spectral profile (sera and PE). Here PC1 is able to segregate between the two classes of biofluids. The corresponding loadings plot shows the metabolites responsible for the discrimination (Additional file 3). Since the ^1^H NMR spectra of the serum and PE were similar (Figure 2), a Discriminant analysis, namely OPLS-DA, was performed in order to tease out the possible subtle differences in terms of concentration of metabolite(s). The OPLS-DA scores plot (Figure 5A) of the serum and PE showed a clear segregation. The R^2 ^X (cum) of this model was 0.76 and Q^2 ^(cum) is 0.85. This signifies considerable distinction between these two classes of fluids. The complementary OPLS loadings of the NMR variables; p(1) and their correlation towards class segregation are presented in Table 2. The OPLS coefficient plot (Figure 5B) showed a marked difference in glucose followed by creatine, glutamine and lipids concentration in serum with respect to PE. The glucose, creatine, glutamine concentration in the PE is higher than that in the serum of the CM mice while lipid concentration is higher in the sera (Figure 5B). Glucose quantification test by the GOD/POD also indicated a significant difference in the glucose levels (p < 0.001; Mann Whitney test) in the serum and PE (Figure 6D). A negative correlation of glucose in the PE with that of serum from MPCA loadings plot suggested that the source of comparatively high amounts of glucose in the PE may derive from the serum (Figure 7B). The concentration of the glucose in the pleural fluid of control mice was found to be 8.9 mM (Figure 6E), consistent with earlier observations [33]. However, the pleural fluid is no more than 5-10 μL. In CM mouse, although the final concentration of glucose in the PE was 7.5 ± 0.7 mM, the volume of the fluid was 200 to 400 μl. Therefore, the entire glucose in PE comes from the serum. Therefore, it appears that the glucose from the serum moves into PE during PE formation in CM mice.

**Table 2:** OPLS-DA loadings [p(1)] and correlation p(corr) of the metabolites towards class segregation (sera and pleural effusion of CM C57BL/6).

Metabolite	Resonance (ppm)	Loading [p(1)]	p(corr)
Lipids	0.88, 1.27, 2.02	0.050, 0.100, 0.040	0.63, 0.66, 0.71
leucine	0.92, 0.94	-0.011, -0.014	-0.46, -0.57
isoleucine	0.99, 1.02	-0.010, -0.012	-0.31, -0.56
valine	1.04	-0.012	-0.41
alanine	1.46	-0.017	-0.48
acetate	1.90	-0.017	-0.62
citrate	2.55, 2.65	-0.018, -0.020	-0.62, -0.65
glutamine	2.44	-0.030	-0.78
pyruvate	2.36	0.020	0.49
creatine	3.03	-0.050	-0.78
lysine	3.01	-0.019	-0.57
choline, GPC, PC	3.20 3.21	0.030 0.030	0.27 0.30
glucose	3.90, 3.82, 3.76, 3.40.	-0.070,-0.077, -0.060, -0.1	-0.88, -0.95,-0.92, -0.93
lactate	4.11	0.07	0.65

GPC-glycerophosphocholine

PC-phosphocholine

In a different set of experiment sera of the CM mice were compared to the sera of uninfected controls by OPLS-DA where glucose seems to be an important molecule for discrimination and was decreased in CM sera (Table 3). This experiment was followed by another experiment where the serum of individual mouse with CM was mixed the corresponding PE of that mouse and then compared to the sera of controls by OPLSDA. The role of glucose as a discriminatory metabolite between these groups decreases i.e pcorr value of glucose in this model decreases (Table 3). This also indicates that pleural effusion causes hypoglycaemia in the host. *Plasmodium *infected RBCs consume glucose at higher rate than the healthy RBCs and hypoglycaemic condition is common amongst severe malaria patients [46,47]. Such a loss of glucose would make the situation more serious. The relative concentration of creatine and glutamine in PE is higher than that of serum as is evident from the OPLS coefficient plot (Figure 5B) and from Figure 6A and 6B respectively, (p < 0.05 for creatine and p < 0.05 for glutamine). On the other hand concentration of lipids in the sera is higher compared to that of pleural effusion (p < 0.05) (Figure 6C). However unlike glucose their correlation towards segregation of PE and serum is not very high (Table 2). Creatine is known to decrease in the serum during malaria due to the increase of creatine phosphokinase (CPK) [48]. Reduced glutamine levels in plasma during malaria are associated with sepsis and mortality rate [49].

**Table 3:** OPLS-DA loadings [p(1)] and correlation p(corr) of glucose towards class segregation A: (sera of CM with control C57BL/6). B: (mixture of sera and pleural effusion of CM with control C57BL/6).

Model	Metabolite	**Loading P **[1]	p(corr)
A (sera of CM and control)	Glucose	-0.25	-0.96
B (Mixture of sera of CM and pleural effusion with control)	Glucose	-0.21	-0.56

This study indicates that cerebral complication of the disease leads to additional 'hypoglycaemia', 'hypocreatine' and 'hypoglutamine' conditions in the blood because of the movement of these components into the PE. This observation also suggests that depletion of glucose, creatine and glutamine in the blood occurs in addition to lactic acidosis, increase of CPK and glutaminolysis, which would add further stress in the patient during CM.

In order to further investigate the correlations of metabolites in the two biological fluids, sera and PE, an MPCA model was generated. PC 1 and 2 captured 59.60 and 24.95% data variation respectively (Figure 7). The eigenvalue (representing a measure of the amount of variance of the score vector) of PC3 onwards was too low, therefore, only the first two PCs were of interest. Since PC2 exhibits homogeneous dispersion of the scores (Figure 7A), the loadings corresponding to this PC were used for further analysis of metabolic inter-compartmental correlations. PC1, on the other hand, mainly shows inter animal variations not related to the disease as it separates one animal from the rest. These loading values of the MPCA of metabolites in serum bear a significant meaning in context to their correlation to form PE. For example, negative loading value of a specific metabolite in serum and its corresponding positive loading value in PE would mean that a decreased concentration of this metabolite in serum is correlated to its increase in PE. This is also valid for the loadings of different metabolites in one particular compartment. From Figure 7B, we see that glucose in the sera is negatively correlated to lactate which indicate glycolysis. Apart from lactate there are positive loadings of pyruvate and alanine in sera. The positive loadings of pyruvate, lactate and alanine with respect to that of glucose in sera indicate the presence of high lactic acidosis in malaria and glucose-alanine cycle operating during CM. The lipids in the sera are negatively correlated to that of lipids in PE; which indicate that lipids in the PE increase with their corresponding decrease from sera. Possibly for lipids/lipoproteins the movement is slow owing to the larger size and so there was a positive loading of lipids in OPLS coefficient plot in sera; indicative of its higher concentration in sera despite its negative loading of MPCA in serum.

The loss of glucose in serum is correlated to increase of glucose in PE, indicating a risk of secondary infection in pleura. However the mechanism behind such transport is not understood. The fate of lactate is just the opposite, i.e. a lower lactate level in PE is correlated to its enhanced level in sera (Figure 7B). A plausible reason could be the need of fast gluconeogenesis in fatal stage of CM.

**Figure 8:**
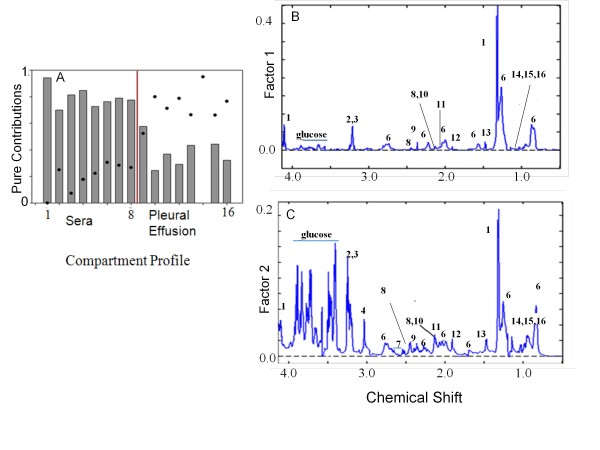
**Compartmental profile of sera and pleural effusion of CM C57BL/6 obtained from MCR-ALS and the normalized pure spectrotype of sera and pleural effusion of CM C57BL/6**. (A) The bars and the dots represent compartmental profile of sera and pleural effusion of CM C57BL/6 mice respectively. (1-8) in the × axis refers to sera while (9-16) refers pleural effusion of the mice arranged in the same order as that of sera. (B) and (C): The key refer to the peaks in Figure 2.

In order to find out whether the two compartments have contributions to each other at the terminal stage of CM, MCR-ALS was performed over these two compartments. Figure 8A shows the pure contribution profile of the two biological fluids. It is apparent that certain changes in the sera co-vary with certain changes in PE and vice versa. Thus the first and the second factor, representing blood serum and PE respectively, also explain a part of PE and sera profile respectively. The pure spectrotype of sera (Figure 8B) is dominated by lactate whereas glucose and creatine are not significant. On the contrary the spectrotype of PE is dominated mainly by glucose followed by creatine and the other metabolites.

## Conclusions

This study reports accumulation of pleural effusion in all CM mice and provides characterization of the same using ^1^H NMR spectroscopy. The metabolic constituents and SDS-PAGE profile of the PE constituents are similar to that of serum, indicating that PE probably originates from the serum. Glucose, creatine and glutamine amounts in the PE are significantly higher than that of the serum. MCR-ALS analysis point towards exchange of constituents between the two bio-fluids. However, an equilibration with respect to metabolite concentration is not seen indicating process(es) other than mere passive diffusion between these two compartments. These results bear important implications to the disease progression mechanism and disease management during CM.

## List of abbreviations

CM: Cerebral Malaria; PE-pleural effusion; CPMG: Carl-Purcell-Meiboom-Gill; NMR: Nuclear Magnetic Resonance; MPCA: Multiway Principal Component Analysis; PCA-Principal Component Analysis; OPLS-DA: Orthogonal Partial Least Square Analysis; MCR-ALS: Multivariate curve resolution: Alternating Least Square; GPC-glycerophosphocholine; PC-Phosphocholine.

## Competing interests

The authors declare that they have no competing interests.

## Authors' contributions

SG carried out all the animal experiments, extracts preparation of organs, NMR data acquisition, processing, statistical modelling and interpretation, and drafted the manuscript. AS was involved in sample preparations, and glucose estimation. SS and HMS conceived the study, helped design the experiments, interpretation of NMR and statistical data and were involved in drafting of the manuscript. All authors read and approved the final manuscript.

## Supplementary Material

Additional file 1 **The SDS-PAGE profile of sera and the pleural effusion of CM mouse**. The total protein concentration of the sera and the pleural effusion was using Bradford reagent. A calibration curve was prepared for the known concentration of protein (BSA). Total protein content was measured from the calibration graph. The significance of the test is done by students t test (Excel-2010). Protein quantification test was done for two categories of samples, sera of the CM mice and the pleural effusion of the CM mice. The samples were mixed with sample buffer and were boiled for 3 mins. Electrophoresis was done in vertical slab electrophoresis BIORAD apparatus. SDS acrylamide gels was used [50] as separating gels (8)% were prepared along staking gel (5%). A molecular weight marker (Sigma) was used for molecular weight calibration. (SDS-200 Carbonic anhydrase-2900, egg albumin-45000, bovine albumin-66000, PhosphorylasecB-97000, β-galactosidase 116000, Myosin 2050000). Click here for file

Additional file 2 **The PCA scores plot of the ^1^H NMR spectra of sera and pleural effusion of mice with CM**. The red and black symbols represent the scores of pleural effusion and the sera of CM C57BL/6 respectively. Click here for file

Additional file 3 **Loadings plot of PCA between ^1^H NMR of sera and pleural effusion of CM C57BL/6**. The loading values on PC1 segregate the sera and pleural effusion CM C57BL/6 mice. Click here for file
