# Supplementary figures and images for: Multivariate modelling with 1H NMR of pleural effusion in murine cerebral malaria
Source: Malar J. 2011 Nov 2;10:330. doi: 10.1186/1475-2875-10-330 (PMC3228806; doi:10.1186/1475-2875-10-330)

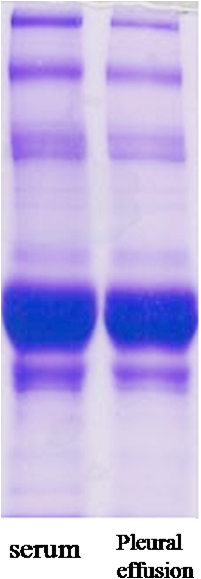

Supplement: Additional file 1 — The SDS-PAGE profile of sera and the pleural effusion of CM mouse. The total protein concentration of the sera and the pleural effusion was using Bradford reagent. A calibration curve was prepared for the known concentration of protein (BSA). Total protein content was measured from the calibration graph. The significance of the test is done by students t test (Excel-2010). Protein quantification test was done for two categories of samples, sera of the CM mice and the pleural effusion of the CM mice. The samples were mixed with sample buffer and were boiled for 3 mins. Electrophoresis was done in vertical slab electrophoresis BIORAD apparatus. SDS acrylamide gels was used [50] as separating gels (8)% were prepared along staking gel (5%). A molecular weight marker (Sigma) was used for molecular weight calibration. (SDS-200 Carbonic anhydrase-2900, egg albumin-45000, bovine albumin-66000, PhosphorylasecB-97000, β-galactosidase 116000, Myosin 2050000). [file 1475-2875-10-330-S1.TIFF]

## Slide 1
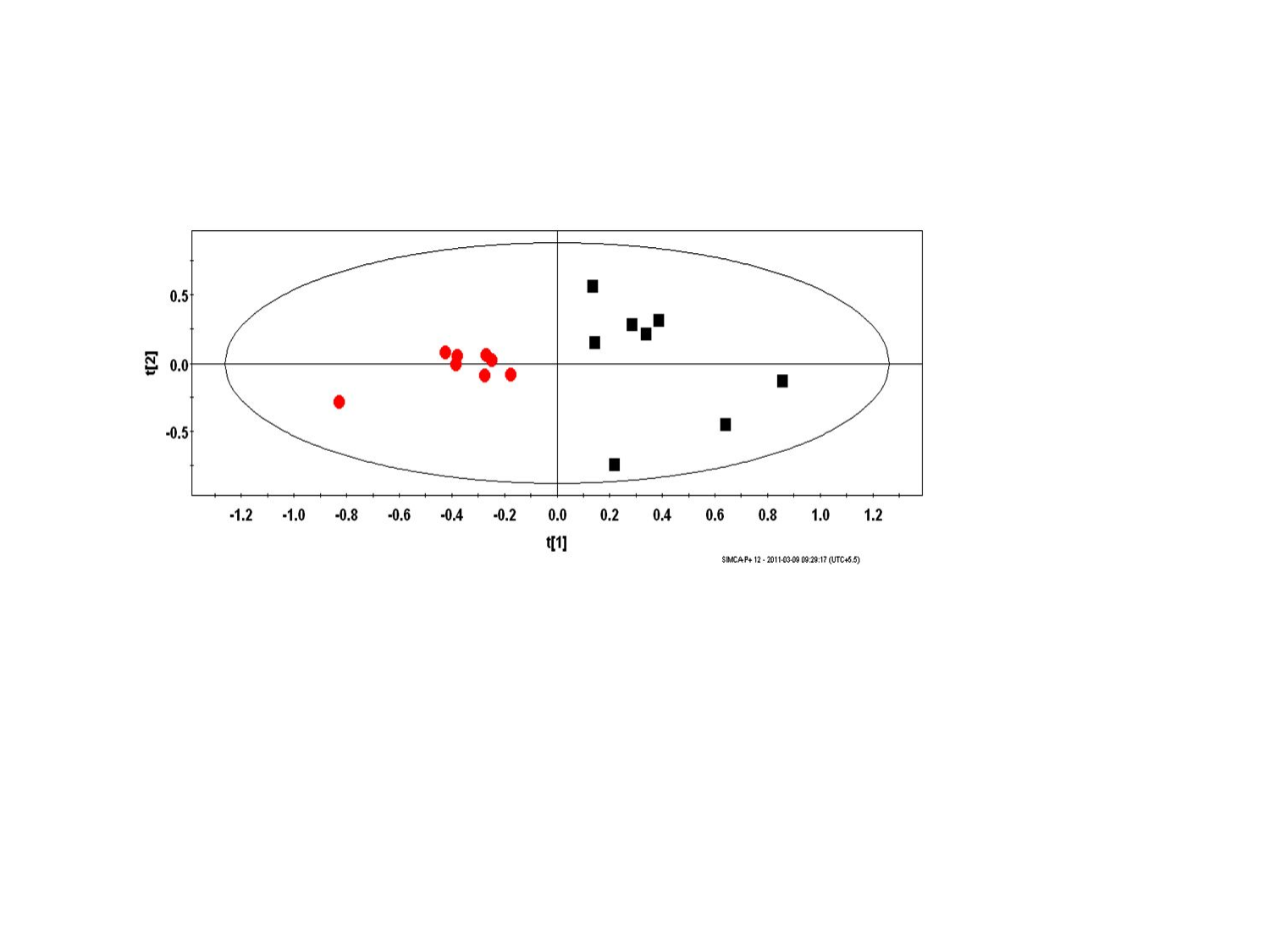

Supplement: Additional file 2 — The PCA scores plot of the 1H NMR spectra of sera and pleural effusion of mice with CM. The red and black symbols represent the scores of pleural effusion and the sera of CM C57BL/6 respectively. [file 1475-2875-10-330-S2.PPT]

## Slide 1
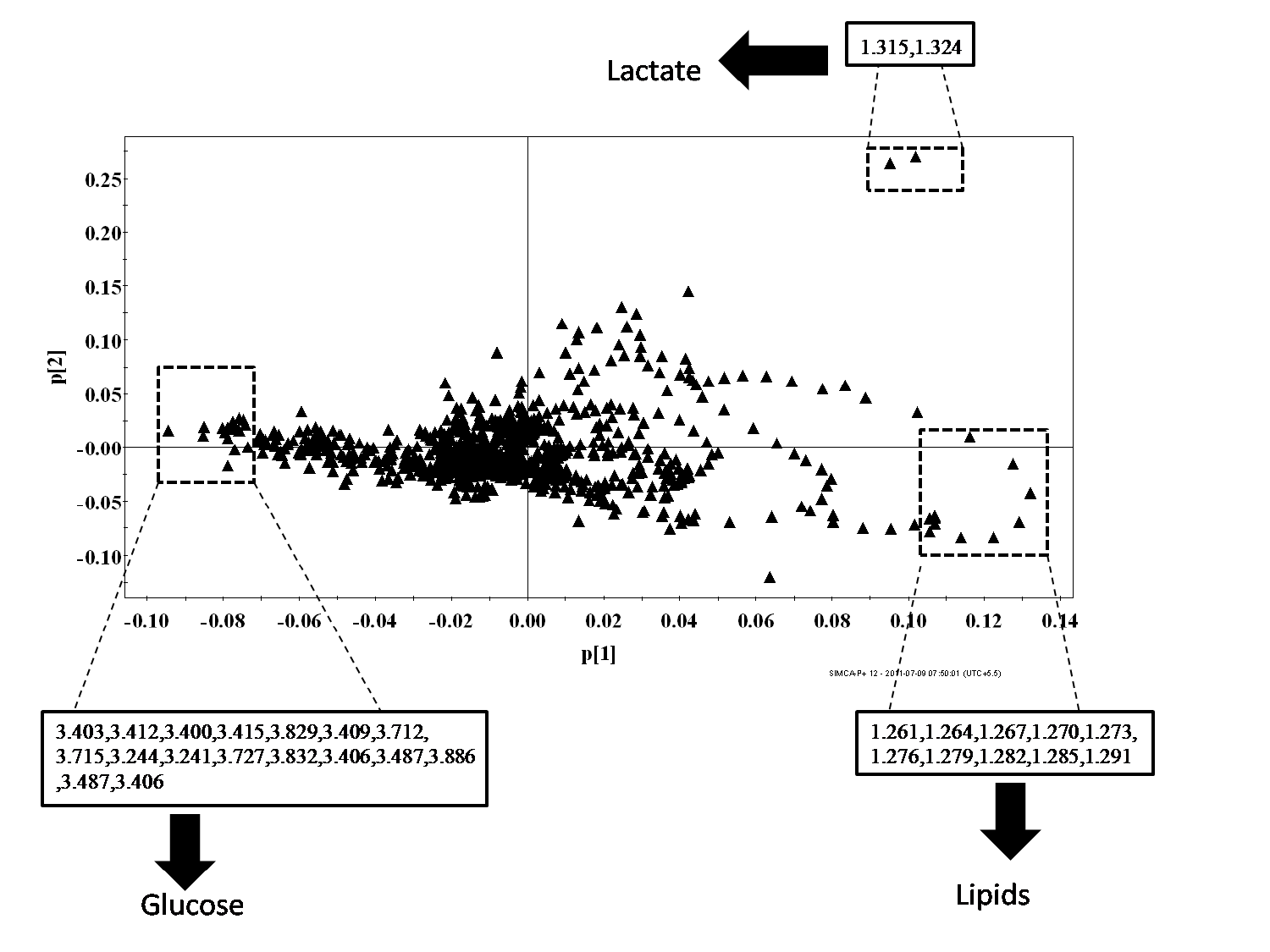

Supplement: Additional file 3 — Loadings plot of PCA between 1H NMR of sera and pleural effusion of CM C57BL/6. The loading values on PC1 segregate the sera and pleural effusion CM C57BL/6 mice. [file 1475-2875-10-330-S3.PPT]
